# Fabrication of High Permittivity Resin Composite for Vat Photopolymerization 3D Printing: Morphology, Thermal, Dynamic Mechanical and Dielectric Properties

**DOI:** 10.3390/ma12233818

**Published:** 2019-11-20

**Authors:** Asish Malas, Dmitry Isakov, Kevin Couling, Gregory J. Gibbons

**Affiliations:** Additive Manufacturing Group, WMG, The University of Warwick, Coventry CV4 7AL, UK; K.Couling@warwick.ac.uk

**Keywords:** additive manufacturing, daylight polymer printing, nanocomposites, photopolymer, dielectric properties, dynamic mechanical properties

## Abstract

The formulation of a high dielectric permittivity ceramic/polymer composite feedstock for daylight vat photopolymerization 3D printing (3DP) is demonstrated, targeting 3DP of devices for microwave and THz applications. The precursor is composed of a commercial visible light photo-reactive polymer (VIS-curable photopolymer) and dispersed titanium dioxide (TiO_2_, TO) ceramic nano-powder or calcium copper titanate (CCT) micro-powder. To provide consistent 3DP processing from the formulated feedstocks, the carefully chosen dispersant performed the double function of adjusting the overall viscosity of the photopolymer and provided good matrix-to-filler bonding. Depending on the ceramic powder content, the optimal viscosities for reproducible 3DP with resolution better than 100 µm were η_(TO)_ = 1.20 ± 0.02 Pa.s and η_(CCT)_ = 0.72 ± 0.05 Pa.s for 20% *w*/*v* TO/resin and 20% *w*/*v* CCT/resin composites at 0.1 s^−1^ respectively, thus showing a significant dependence of the “printability” on the dispersed particle sizes. The complex dielectric properties of the as-3D printed samples from pure commercial photopolymer and the bespoke ceramic/photopolymer mixes are investigated at 2.5 GHz, 5 GHz, and in the 12–18 GHz frequency range. The results show that the addition of 20% *w*/*v* of TO and CCT ceramic powder to the initial photopolymer increased the real part of the permittivity of the 3DP composites from ε’ = 2.7 ± 0.02 to ε’_(TO)_ = 3.88 ± 0.02 and ε’_(CCT)_ = 3.5 ± 0.02 respectively. The present work can be used as a guideline for high-resolution 3DP of structures possessing high-ε.

## 1. Introduction

Additive Manufacturing (AM), also known as three-dimensional printing (3DP), has shown the potential to fabricate components with tailored and complex designs and is being adopted in various industrial domains. The American Society for Testing and Materials (ASTM) classified AM into 7 standard processes [1]. Among these, material extrusion (fused deposition modelling, FDM) [2], vat photopolymerisation (stereolithography, SLA) [3] and material jetting (multi-jet modelling, MJM) [4] are commonly adopted by researchers due to affordability, easy customisation and wide possibilities for application.

Various 3DP processes have been extensively used in the fabrication of optical [5], THz [6,7], microwave and RF dielectric elements and devices [8,9,10]. For such applications, the dielectric properties of the components are essential for a predictable performance. Whilst commercial off-the-shelf feedstock AM polymer materials provide a very limited range of dielectric characteristics [11], the customization of the feedstock precursor by adding high-dielectric permittivity (high-ε) ceramic powder may significantly vary the accessible values of dielectric parameters [12], thus expanding the possibilities and applications of 3DP [13,14,15].

The present work was mainly motivated by the need for a material exhibiting high dielectric properties suitable for 3DP of devices for high-frequency applications, targeting the GHz–THz range. While, as shown in the example above, material extrusion can provide feedstock with dielectric permittivity, ε, as high as 11, and feature resolution is typically within the 100–200 µm range. In the present study, the Digital Light Processing (DLP, variation of SLA) was chosen due to its ability to provide smooth surface finishes [4] and high spatial resolution of 20 µm or better [16,17], therefore providing satisfying conditions for 3DP of complex structures with a sub-wavelength (<λ/5) element size. This technology comprises photopolymerization through cross-linking by a light source (VIS, or UV laser or LED) of a liquid mixture of photo-reactive monomer and photo-initiator [18].

Recently, the combination of structural (polymers) and functional (conducting and high permittivity ceramic fillers) materials has been investigated to achieve multifunctional 3DP parts with good structural stability [19]. Furthermore, by incorporating additives such as organoclay, carbonaceous nano-fillers (carbon nanotubes (CNTs), graphene based fillers, carbon black (CB), carbon nano-fibres (CNF) and metallic nanoparticles), it is feasible to fabricate 3DP materials with advanced target properties and multi-functionality superior to those of unfilled 3DP materials, which broadens the area of application [20,21,22]. Gonzalez et al. [23] used the DLP technique to 3D print a series of objects using blends of photopolymer, photo-initiator and CNTs. They observed a decrease in the mechanical performance of the 3DP photopolymer-CNT composites due to a slight decrease in crosslinking density as a result of the incorporation of CNTs. However, they found that the electrical conductivity of the 0.1% *w*/*w* CNT-loaded composite increased almost 3 orders of magnitude compared to the pure photopolymer formulation.

Weng et al. [24] have observed that the incorporation of 5% *w*/*w* of SiO_2_ nano-powder into a commercial SLA feedstock increased the tensile strength and modulus of the 3DP samples by 20.6% and 65.1% respectively. Lin et al. [25] 3D printed an SLA nanocomposite using a photopolymer filled with 0.2% *w*/*w* graphene oxide leading to a 62.2% increase of the tensile strength. Manapat et al. [26] reported 673.6% increase in the tensile strength for the 3DP composite after adding 1% *w*/*w* graphene oxide; similarly, adding 10% *w*/*w* multi-walled CNT into the SLA precursor resulted in 7.5% improvement in the tensile strength [27]. The dielectric properties of a 3DP acrylic ester resin and 1.5% *w*/*w* CNT composite in the 4—18 GHz frequency range [28] revealed approximately two times increase in permittivity, although showing high dielectric losses.

Although there has been prior research in 3DP of structurally enhanced photopolymer/ceramic composites, there has been little investigation of Vat Polymerisation polymer/composites potentially suitable for novel electromagnetic applications, where the combination of high dielectric permittivity, low-dielectric loss, and good spatial resolution is essential. It should be noted that the application of 3DP technologies for fabrication of dielectric devices for frequencies above 20 GHz remain almost an unexplored area. Otter et al. [29] have demonstrated the future potential of high-resolution SLA by 3DP of a rectangular waveguide from acrylic plastic followed by metal coating, thus offering the benefits of lightweight rapid prototyping at very low cost when compared with traditional manufacturing. Metal coating of an SLA 3DP optical device has been also explored by Fullager et al. [30]. It demonstrated very similar performance to an identical commercially available off-axis paraboloid in the THz spectral range. Duangrit et al. [31] reported on digital light-processing (DLP) SLA and polymer-jetting 3DP using different photopolymers and investigated their suitability in terms of printing resolution and material characteristics for mm-wave and THz applications. They reported that typical photopolymers for these technologies have dielectric constants between 2.0 and 3.1, and the dielectric loss tangents are from 0.008 to 0.102 for 3DP samples in the 0.2–1.4 THz. Several devices, including gradient index devices and electromagnetic crystals have been 3D-printed using polymer jetting and characterised in the 30–350 GHz frequency range [32]. A potential of using SLA for the fabrication of VIS-optically transparent devices using photocurable silica nanocomposite has also been shown [33].

Here, we focus on the development of a feedstock precursor with a high dielectric constant for high-resolution low-cost Daylight Polymer Printing (DPP) technology. Nano- and micro-powders of TiO_2_ (TO) and CaCu_3_Ti_4_O_12_ (CCT) are used as a high-ε ceramic fillers with the objective of achieving significant improvement in the dynamic mechanical, thermal, and dielectric properties of the DPP 3DP composites, as compared to off-the-shelf commercial feedstock, and to potentially increase the range of applications of this process.

## 2. Materials and Methods

### 2.1. Preparation of the Composite Photopolymer Precursor

To formulate the photopolymer composite dispersions and to enhance the distribution of the ceramic powder in the photopolymer composite dispersion, a dispersing agent, DISPERBYK-2055 (BYK-Chemie Gmbh, Wesel, Germany) was added to the photopolymer (daylight precision firm resin (black) (Photocentric Ltd., Peterborough, UK)) in a 100 mL amber glass bottle and stirred (1000 rpm) on a magnetic stirrer at RT for 1 h. After that, the filler (nano/micro-sized) was added to the previous solution and magnetically stirred (1000 rpm) at RT for 6 h and ultra-sonicated for 2 h. Fillers employed were titanium (IV) oxide (TiO_2_), nanopowder, <100 nm (Sigma-Aldrich, Dorset, UK) and calcium copper titanate (CaCu_3_Ti_4_O_12_), ~3–5 µm (Thermograde Process Technology Ltd., Stone, UK). Photopolymer containing 5, 10, 20% *w*/*v* filler (TO and CCT) along with 2% *w*/*w* dispersing agent (with respect to filler weight) composites were formulated.

**Figure 1:**
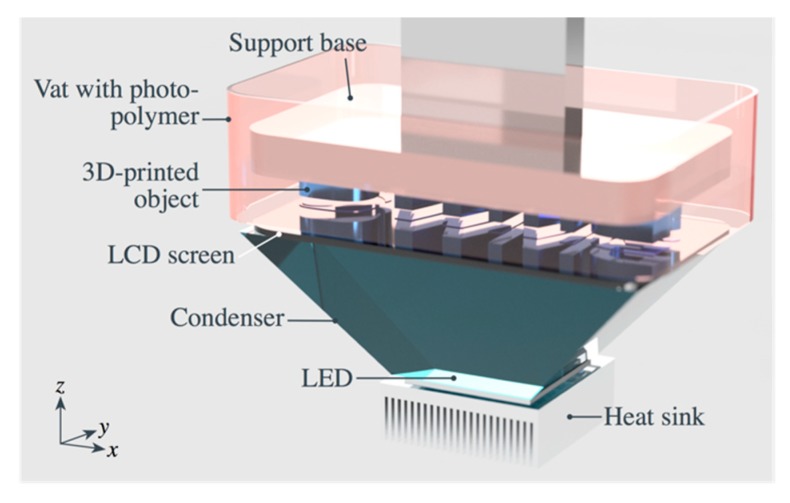
Schematic of the daylight LCD 3D printer. The photopolymer is cured at the bottom of the vat by the VIS blue light emitted by the LED source and masked through the LCD screen.

A Liquid Crystal Precision (LCP) 3D Printer (Photocentric Ltd., Peterborough, UK) was used for the DPP 3DP of the formulated photopolymer. A diagram of the LCP 3D printer is shown in Figure 1. Contrary to conventional SLA [3,16], where solidification of the photopolymer occurs under exposure of a focused source of UV light, the LCP 3D printer uses a low-power LED array as a source for the blue light (400 nm wavelength), which is passed through a thin LCD panel, which works as an optical shutter for the light source, thus providing whole-layer exposure. The typical 2K resolution of the LCD provides an XY pixel size of 42 µm (600 dpi), over a build volume of 12.1 × 6.8 × 16 cm^3^ with 25 µm layer thickness. In the DPP 3D printer, a layer thickness of 100 µm and an exposure time of 80 s for each layer was used to 3D print the composites and un-filled photopolymer. To increase the crosslinking in the formulated composites and obtain a consistent 3D printing, the exposure time was extended to 100–110 s.

### 2.2. Characterisation Techniques

#### 2.2.1. Vat Viscosity Measurements

Dynamic viscosity of the formulated photopolymers was measured using a HAAKE Mars III Rheometer (ThermoFisher Scientific, Glouster, UK) and 25 mm parallel plates at RT with a shear rate range of 0.01–1000 s^−1^.

#### 2.2.2. Structural Characterisation

X-ray diffraction (XRD) spectra of the two fillers were recorded using an Empyrean X-ray Diffractometer (Malvern Panalytical Ltd., Malvern, UK) with a CoKα source at a generator voltage of 40 kV, 40 mA current, wavelength of 1.79 Å, scan range of 10 to 110° and step size 0.01° at RT.

Scanning electron microscopy (SEM) imaging was performed using a Zeiss Sigma FE SEM (Carl Zeiss Microscopy Ltd., Cambourne, UK) on powder samples which were sputter coated using an Au/Pd target.

Surface topography of the 3DP composites was obtained using a 3D Optical Profiler (Bruker ContourGT-X, Bruker UK Ltd., Coventry, UK), white light interferometer with 50× optical lens. SEM imaging was carried out using a JEOL-SEM and Zeiss Sigma FE SEM (Cambourne, UK) on fractured samples, which were sputter coated using an Au/Pd target.

#### 2.2.3. Thermal Dynamic Characterisation

Thermal degradation behaviour of the 3DP composites was investigated using a TGA1-STARe Thermogravimetric analyser (TGA) (Mettler-Toledo Ltd., Leicester, UK) in the temperature range 25–550 °C, at a heating rate of 10 °C/min under nitrogen atmosphere. Differential Scanning Calorimetry measurements were carried out using a DSC 1 Stare (Mettler-Toledo Ltd., Leicester, UK) between −40 °C and 200 °C with a heating and cooling rate of 10 °C/min.

Dynamic Mechanical Thermal Analysis (DMTA) was performed on samples 5 mm × 9 mm × 1 mm in single cantilever mode with a 50 µm amplitude, a frequency of 1 Hz and a heating rate of 5 °C/min in the temperature range of −30–200 °C using a Tritec 2000 DMA (Mettler-Toledo Ltd., Leicester, UK). The frequency dependence of storage modulus (G′), loss modulus (G″) and loss factor (Tan δ) were measured for the same dimension samples in single cantilever mode with a 50 µm amplitude at 25 °C in the frequency range of 0.01–50 Hz.

#### 2.2.4. Dielectric Characterisation

The complex dielectric properties of the 3DP composites were obtained using a 2-port Vector Network Analyzer (Keithley E5063A, Keithley, Cleveland, USA) at 2.5 GHz and 5 GHz with the split-post dielectric resonator (SPDR) technique and in the 12–18 GHz frequency range using the waveguide technique. When using the waveguide technique, the SPDR was used for accurate measurements of the dielectric loss [34]. The SPDR technique allows the determination of complex permittivity with greater sensitivity than Thru-Reflect-Line (TRL) methods. The samples were printed as 30 mm diameter, 1.5 mm in thick flat discs with and 16 mm × 8 mm × 2 mm rectangular blocks, suitable for insertion into the waveguide transmission/reflection line. The waveguide technique involved measuring the two-port complex scattering reflection S_11_, S_22_, and transmission S_21_ and S_12_ parameters, followed by retrieving the relative complex permittivity ε*_r_* using the Nicholson–Ross–Weir method [35,36].

## 3. Results and Discussion

### 3.1. Precursor Characterization

**Figure 2:**
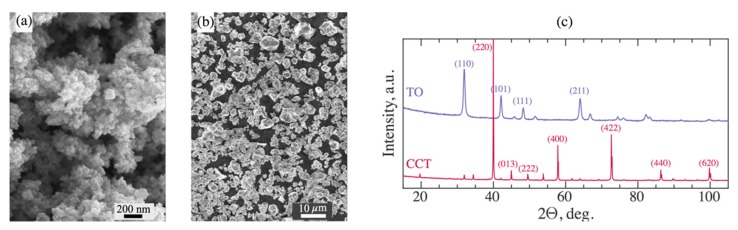
SEM images of raw titanium oxide (TO) (**a**) and calcium copper titanate (CCT) particles (**b**); and (**c**) XRD patterns of corresponding TO and CCT nano- and micro-particles.

TO nanoparticles are spherical and agglomerated, while CCT particles show polyhedral morphology with a particle size of 3–5 µm (Figure 2a,b). The crystal structures of the TO and CCT powders are shown in Figure 2c. The XRD spectrum of TO exhibits prominent rutile peaks at (110) and prominent anatase peaks at (101) and (211) at 2θ = 32°, 42° and 64° respectively, and therefore can be classified as a tetragonal structure, P42/mnm space group [37,38]. The XRD spectrum of CCT shows pronounced peaks at (110), (211), (220), (013), (222), (123), (400), (422), (440) and (620) at 2θ = 19.7°, 34.5°, 40°, 45°, 49.6°, 53.8°, 57.9°, 72.7°, 86.4° and 99.9°, respectively, thus revealing its cubic body-centred perovskite-related structure, Im-3 space group [39,40].

**Figure 3:**
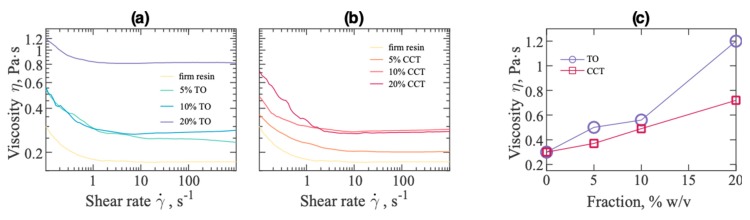
Viscosity of formulations of (**a**) TO composite and (**b**) CCT composite as a function of the shear rate and (**c**) filler content (at shear rate of 0.1 s^−1^) at ambient temperature.

Figure 3 shows flow behaviour, i.e., shear rate dependence of viscosity of the liquid precursor composite photopolymer with dispersed TO and CCT powders. Dispersants have the unique ability to control the colloidal interactions by replacing the powder/powder and powder/air interfaces with a powder/binder interface and hence control the viscosity of the suspension [41,42]. The addition of dispersant enhances the ceramic particle loading in the photo-resin by enhancing the distribution of the ceramic powder in the photo-resin composite suspension i.e., in the presence of dispersant in the resin formulation, the particle ratio loading in the photo-resin can be increased without significantly compromising the viscosity of the formulated resin composites. Additionally, the dispersant stabilized the TO and CCT particles in the photopolymer against sedimentation which is required for the DPP process to successfully 3DP. Inhomogeneity nor before or after the printing have been observed. All composites exhibited shear thinning behaviour. As expected, for all studied dispersions (Figure 3a–c), the viscosity increased with filler content. As seen in Figure 3a,c, the viscosity of the TO-filled photo-resin suspension gradually increased with an increase in TO content, and the viscosity of the 5, 10 and 20 % *w*/*v* TO increased from 0.30 Pa.s (unfilled photo-resin at 0.1 s^−1^) to 0.50 ± 0.02, 0.56 ± 0.02 and 1.20 ± 0.02 Pa.s at 0.1 s^−1^, respectively. It can be observed from Figure 3b,c that the viscosity of 5, 10 and 20% *w*/*v* CCT composites increased to 0.37 ± 0.05, 0.49 ± 0.05 and 0.72 ± 0.05 Pa.s at 0.1 s–1, respectively. The increase in viscosity with increasing filler content is higher for the TO nano-composite than for the CCT micro-composite (Figure 3c), which indicates that TO has a stronger interaction with the photopolymer than it does with CCT. The viscosity of the photo-resin formulation is an important parameter to achieve good performance in SLA [43]. For a successful DPP process, the ceramic suspension should have a viscosity range between 2–5 Pa.s in order to achieve complete layer recoating. In this work, the viscosities of the formulated photo-resin composites (5, 10 and 20 wt% particle loaded) were well within the viscosity range required to achieve better 3DP performance that resulted in successful 3DP of the formulated composites.

The rheological properties of neat photopolymer can also be affected by the size of the filler particles. Usually, the viscosity of composites containing smaller particles (nanometre range) is greater than the viscosity of composites filled with larger particles (micrometre range) at the same volume fraction [44,45].

Faitel’son et al. [46] prepared low-density polyethylene (LDPE)/calcium metasilicate composites and studied the effect of filler on the viscosity of the composites. They observed gradually decreasing shear viscosity with an increase in filler loading and concluded that the decrease in shear viscosity was attributed to the aggregation of the filler particles at higher loading and binding of part of the matrix in the aggregations. Most studies have observed an increase in viscosity with filler volume. Starr et al. studied the effect of filler particle clustering on the rheology of a polymer melt and noticed that the viscosity of the composite increases with increasing dispersion of the filler particles [47]. D. A. Komissarenko et al. [48] prepared acrylate-based suspensions containing zirconia fillers in the presence of different surfactants for DLP 3D printing. They observed shear thinning rheological behaviour and increase in the viscosity for the filled composites. Gojzewski et al. [49] formulated acrylate/silica dispersions and photo-cured composites. They reported that the viscosity of the formulated composition increases with the silica content. Figure 3c exhibits the trend of increased viscosity of the photo-resin composites suspension with increased filler loading, which is expected and in good agreement with the literature.

### 3.2. Characterization of 3D Printed Samples

#### 3.2.1. Surface Structure of 3D Printed Photopolymer Composites

**Figure 4:**
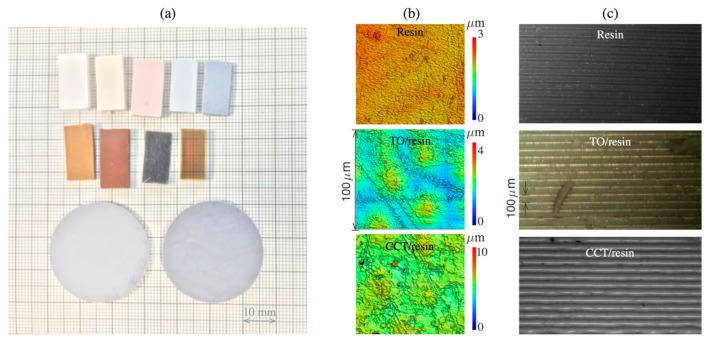
(**a**) Examples of the 3D-printed samples with various content of the dielectric ceramic fillers (smallest square of the background represents 1 mm). The colour and transparency of the samples are the function of loaded particles and grade of the photopolymer. The rectangular-shape blocks were printed to be fitted into the waveguide flange while disks were used for the split-post resonator. (**b**) Topography of the as-printed top surface (xy-plane) for 3DP un-filled resin and TO and CCT filled 10% *w*/*v* composites. (**c**) Side (zx-plane) of the as-printed samples showing the build layers.

Figure 4 shows various samples 3D printed using the formulated precursor photopolymers. The shape and size of the printed samples have been chosen in accordance with requirements for dielectric measurements, i.e., either as a thin disk or as a rectangular block. Figure 4b,c also shows the close-up topographical features on the xy (top-surface) (b) and zx (side) (c) planes. As expected, the average surface roughness (Sa) of the 10% *w*/*v* TO (0.18 ± 0.02 µm) and CCT (0.32 ± 0.02 µm) 3DP composites is higher than that of the 3DP un-filled photopolymer (0.11 ± 0.02 µm), due to the presence of dispersed filler particles.

**Figure 5:**
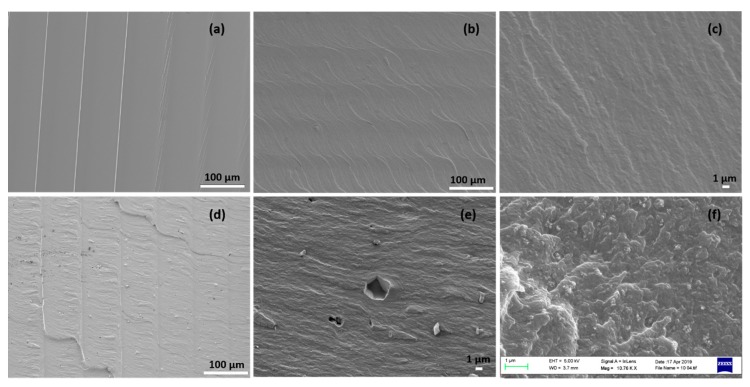
SEM images of the fractured surface of the DPP 3DP (**a**) un-filled photopolymer, (**b**–**c**) 10% *w*/*v* TO composite (low and high magnifications), (**d**–**e**) 10% *w*/*v* CCT composite (low and high magnifications); and (**f**) state of dispersion of TO in the 10% *w*/*v* TO/photopolymer composites.

Figure 5a–e present the SEM micrographs of the fracture surfaces obtained in the un-filled photo-polymer and TO/CCT 3DP composites. The fractural surface of the 3DP photopolymer are smooth, while the cross-sections of the composites exhibit a rough and tortuous path, due to the dispersed TO and CCT particles modifying the crack lines depending on their orientation in the polymer matrices. For the micron sized CCT composites, the compatibility between filler and resin was not ideal, compared to the nano-sized TO composites. Some CCT particles (Figure 5e) were directly pulled out of the photopolymer matrix. Figure 5f is a close-up of the dispersion of TO nanoparticles in the photopolymer, 10% *w*/*v*.

#### 3.2.2. Dielectric Properties of the 3D Printed Photopolymer Composites

**Figure 6:**
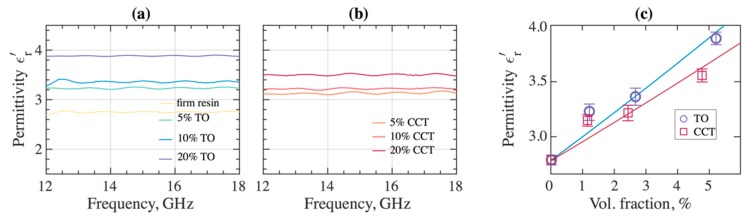
The relative permittivities of the DPP 3DP samples with dispersed (**a**) TO and (**b**) CCT ceramic powder measured by the conventional waveguide method. (**c**) The corresponding average values of the dielectric permittivity as a function of volume fraction (note, that (**a**,**b**) shows the value for wt/v%) and solid curves represent the fitting of the experimental values by the effective medium theory [50].

Figure 6 shows the measured real part of the relative dielectric permittivity as a function of frequency for 3DP TO and CCT composites and photopolymer as a reference for the 12–18 GHz frequency range. The addition of the ceramic particles to the photopolymer increases the permittivity of the composites, reaching ε′(TO) = 3.88 ± 0.02 and ε′(CCT) = 3.5 ± 0.02 for 20% *w*/*v* of TO and CCT, respectively. In general, the addition of TO appeared to be a more effective route for increasing the relative dielectric permittivity of the composites.

The results of dielectric permittivity measurements obtained by the SPDR method for nominal 5 GHz are consistent with the waveguide measurements and show the complex dielectric permittivity of the 3DP un-filled photopolymer as ε′(resin) = 2.89 − j0.0197, while for the 3DP 20% *w*/*v* TO composites, the permittivity is ε′(TO) = 3.85 − j0.0321. The results of dielectric permittivity in TO-based 3DP samples are in good agreement with the effective medium theory [50] and experimentally observed results for TO/rubber composites [51]. Figure 6c shows the real part of the permittivity of 3DP composites for 5, 10 and 20% *w*/*v* volume fractions of TO and CCT. The predicted values were calculated in accordance with [50] (Equation (1)) taking into consideration that TO are nearly spherical particles (*n* = 0.1 correction factor to compensate for the shape and diameter of the fillers) and CCT are less spherical (*n* = 0.12):

(1) $$\varepsilon_{eff}=\varepsilon_{m}\left(1+\frac{f\left(\varepsilon_{f}-\varepsilon_{m}\right)}{\varepsilon_{m}+n\left(1-f\right)\left(\varepsilon_{f}-\varepsilon_{m}\right)}\right)$$

where *ε_eff_*, *ε_m_*, and *ε_f_* are the effective real part of the dielectric constant of the composite, polymer matrix, and ceramic filler, respectively, and *f* and *n* are the volume fraction and the correction. The permittivities *ε_f_* of the TO and CCT ceramic filler were stood 100 and 65 respectively. Surprisingly, CCT-filled samples show very moderate increase in permittivity in spite of the reported giant dielectric constant in this material [52,53]. It is likely that the synthesis parameters, such as high-temperature annealing of the as-prepared ceramic and the particles size, would dramatically affect the dielectric permittivity [54]. As in our case, the CCT ceramic powder was used as-is without post-synthesis treatment.

#### 3.2.3. Thermal Analysis of the 3D Printed Photopolymers

**Figure 7:**
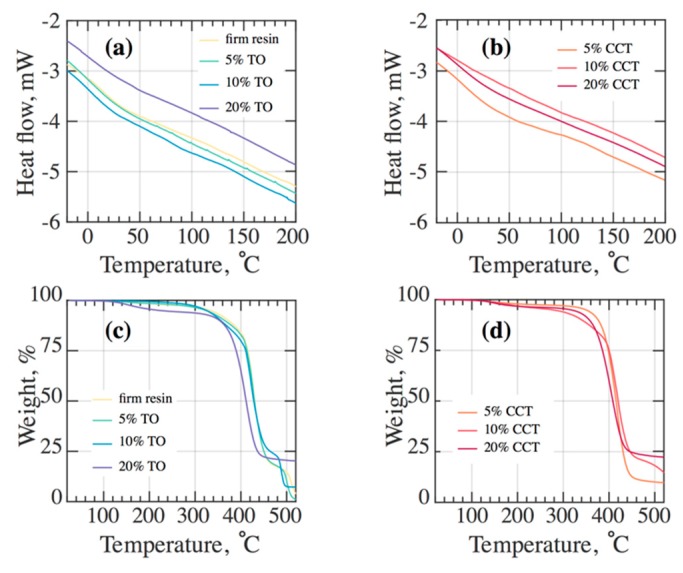
DSC thermograms of 3D printed (**a**) TO composite, (**b**) CCT composite and TGA curves for the 3DP (**c**) TO composite, (**d**) CCT composite.

Differential scanning calorimetry (DSC) was performed for the 3DP TO and CCT composites to observe the effect of different fillers on their glass transition temperature, T_g_. Figure 7a,b shows the DSC thermograms of the composites loaded with 5, 10 and 20% *w*/*v* ceramic fillers. The peaks corresponding to the T_g_ were not very prominent, so the values for the glass transition temperature were extrapolated from the cross-section point of two tangents. There were no melting and crystallisation peaks observed, which confirms the amorphous structure of all the 3DP samples. The significant increase in T_g_ of the 3DP composites is attributed to the reinforcing effect of the fillers that hindered the mobility of the polymer chains [55].

The thermal degradation of the 3DP photopolymer and its composites presented in Figure 7c,d reveals that the onset degradation temperature (T_0_) and maximum degradation temperature (T_max_) were decreased for the composites compared to the un-filled photopolymer. Furthermore, T_0_ gradually decreased with an increase in the composite’s filler content. This may be due to the decrease in molecular weight during photo-polymerization (DPP 3DP process) in the presence of fillers [56]. The primary explanation for the induction of thermal instability in the 3DP composites is postulated to be due to the improved and effective heat transfer to the photopolymer phase through the dispersed ceramic fillers [57]. Table 1 summarises the thermal properties of the 3DP composites.

#### 3.2.4. Dynamic Mechanical Analysis of the 3D Printed Photopolymer and Its Composites

**Figure 8:**
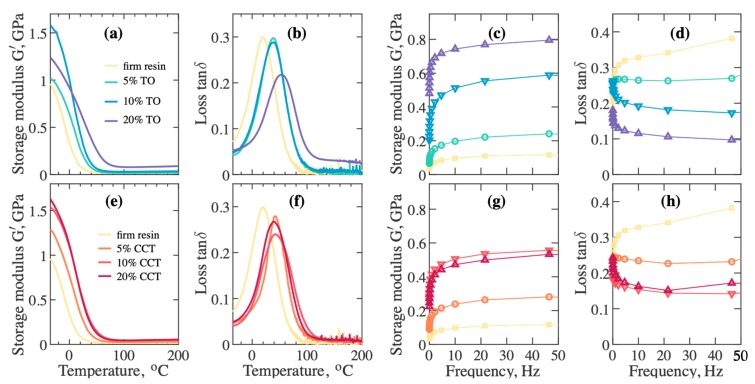
Temperature dependent storage modulus, G’, for 3DP (**a**) TO composite, and (**e**) CCT composite; loss factor tanδ, for (**b**) TO composite, and (**f**) CCT composite; frequency dependent G’ for 3DP (**c**) TO composite, and (**g**) CCT composite; and tanδ for (**d**) TO composite, and (**h**) CCT composite.

Under the application of periodic mechanical energy, the elastic segment of any viscoelastic material stores energy, whereas the viscous part depletes energy in accordance with the hypothesis of viscoelasticity [58]. The storage modulus G is the complex measure of the stored energy per cycle of the applied sinusoidal stress and it measures the elastic response of the materials. The temperature dependency of the real part G′ and loss tangent tanẟ for the different composites having varying TO and CCT content is shown in Figure 8a,b,e,f. G′ sharply declines for all 3DP samples in the primary relaxation process (glass transition) where tanẟ goes through a maximum followed by a rubbery plateau region. The increase of G′ with the increase in the TO content is due to the reinforcing potential of the dispersed TO nanoparticles in the photopolymer. The exfoliation of nano-sized TO in the polymer matrix, along with the adhesion between photopolymer and dispersed TO, significantly enhances the interfacial area, which increases the reinforcement capability (as seen in Figure 5c,f, there is no evidence of pull out of TO particles from photopolymer matrix indicating good interfacial adhesion between particles and matrix). The storage modulus for the 3DP 20% *w*/*v* CCT composites also significantly increased at 25 °C (typical operational temperature of the 3DP) compared to the pure photopolymer (Figure 8e). However, in the contrast with the TO case, the 20% *w*/*v* CCT composite exhibited a lower storage modulus at ambient temperature compared to the 10% *w*/*v* CCT composite, which may be due to the agglomeration of CCT in the polymer matrix that resulted in a moderate reinforcing effect exerted by the filler at higher loading.

The mobility of the polymer chain segments and free volume primarily affect the thermomechanical properties of a polymer when it is cooled down and put into the glassy state [59]. The 3DP TO and CCT composites have higher glass transition temperature, T_g_, than that of the un-filled photopolymer (Figure 8b,f). T_g_ of the 20% *w*/*v* 3DP TO composite was increased by 33 ± 3 °C, compared to the T_g_ of 3DP un-filled photopolymer.

Figure 8c,d,g,h show the storage modulus G’ and loss of the 3DP TO and CCTO composites as a function of frequency. The dispersion curve of G′ represents the frequency response of elasticity. The storage modulus for the composites increased steeply with an increase in frequency. The behaviour of tanẟ vs frequency signifies the frequency response of the viscous behaviour of polymeric materials. The increase in G′ for the 3DP composites may result from the increase in molecular chain rigidity, due to the restriction of the mobility of the chain segments by the dispersed fillers and the interfacial adhesion between the polymer chains and fillers [60]. A steep decrease in tanδ with an increase in the frequency also indicates an elastic nature of the samples. The frequency response of tanδ (Figure 8d,h) for the 3DP composites exhibited a stable damping property within the measured range of frequency. It was also noticed that G′ increases and tanẟ decreases with an increase in filler content. The mobility of the photopolymer chain segment was highly restricted by the filler particulates and resulted in a decrease in the loss factor.

## 4. Conclusions

The present research explores the high resolution DPP 3DP of photopolymer composites containing TO and CCT particles based on visible light curing technology for high frequency electromagnetic applications. Composites filled with 5, 10 and 20% *w*/*v* of TO and CCT were formulated and the shear viscosity of the formulated composites resin was carefully studied. XRD analysis confirmed the tetragonal structure of TO and body-centred cubic perovskite structure of CCT. The surface topography of the 3DP composites shows that the surface roughness of the TO composites was highly comparable to the un-filled 3DP photopolymer due to better dispersion of the nano-sized TO in the polymer matrix. SEM analysis of the fractured surface of the 3DP composites exhibited a rough and tortuous fractured surface, compared to the smooth fracture surface of the 3DP un-filled photopolymer. The maximum thermal degradation temperature for the composites was decreased compared to the un-filled photopolymer, which may be due to the decrease in molecular weight of the photopolymer during photo-polymerization in the presence of fillers. On the contrary, the glass transition temperature, Tg, and the real part of the storage modulus, G, increased for the composites, showing higher values for 20% *w*/*v* TO, due to the better dispersion and reinforcing effect of the nano-sized TO compared to the micro-sized CCT. The real part of the dielectric permittivity was ε′_(TO)_ = 3.88 ± 0.02 and ε′_(CCT)_ = 3.5 ± 0.02 for the 20% *w*/*v* TO and 20% *w*/*v* CCT composites, compared to ε′_(photopolymer)_ = 2.67 ± 0.02, and showed no dispersion in the 2.5–18 GHz frequency range. Such dielectric properties may be attractive for 3DP of advanced electro-magnetic devices for high-frequency communications, where high spatial resolution of the feature elements is essential. This paper can also be used as a guideline for the formulation of bespoke precursors with tailored properties (e.g., conductive, magnetic, optical) for DPP 3DP, with the optimal viscosities for reproducible 3DP in the range from 1.20 to 0.72 Pa.s for nano- and micro-sized functional inclusions, respectively.

## Figures and Tables

**Table 1:** Glass transition temperature T_g_, onset degradation T_0_, and maximum degradation temperature T_max_ obtained in the 3D-printed samples.

Samples	T_g_ (°C)	T_0_ (°C)	T_max_ (°C)
Un-filled resin	28.0 ± 3.4	324 ± 3.8	428 ± 2.8
5% TO	39.5 ± 3.7	322 ± 2.9	418 ± 3.0
10% TO	37.0 ± 2.9	305 ± 3.2	420 ± 3.5
20% TO	43.0 ± 3.1	301 ± 2.8	405 ± 3.1
5% CCT	39.0 ± 3.2	295 ± 3.0	428 ± 2.7
10% CCT	36.0 ± 3.9	284 ± 3.0	422 ± 3.0
20% CCT	35.0 ± 3.5	281 ± 3.2	409 ± 2.9

## References

[1] ISO/ASTM 52900: Additive Manufacturing—General Principles—Terminology. https://www.iso.org/obp/ui/#iso:std:iso-astm:52900:ed-1:v1:en.

[2] Live Science. https://www.livescience.com/39810-fused-deposition-modeling.html.

[3] Live Science. https://www.livescience.com/38190-stereolithography.html.

[4] Gibson I., Rosen D., Stucker B. (2010). Additive Manufacturing Technologies.

[5] Assefa B.G., Pekkarinen M., Partanen H., Biskop J., Turunen J., Saarinen J. (2019). Imaging-quality 3D-printed centimeter-scale lens. Opt. Express.

[6] Vogt D.W., Anthony J., Leonhardt R. (2015). Metallic and 3D-printed dielectric helical terahertz waveguides. Opt. Express.

[7] Van Putten L.D., Gorecki J., Fokoua E.N., Apostolopoulos V., Poletti F. (2018). 3D-printed polymer antiresonant waveguides for short-reach terahertz applications. Appl. Opt..

[8] Wu Y., Grant P.S., Isakov D. (2018). 3D-printed λ/4 phase plate for broadband microwave applications. Opt. Express.

[9] Isakov D., Stevens C.J., Castles F., Grant P.S. (2016). 3D-Printed High Dielectric Contrast Gradient Index Flat Lens for a Directive Antenna with Reduced Dimensions. Adv. Mater. Technol..

[10] Monkevich J.M., Le Sage G.P. (2019). Design and Fabrication of a Custom-Dielectric Fresnel Multi-Zone Plate Lens Antenna Using Additive Manufacturing Techniques. IEEE Access.

[11] Deffenbaugh P.I., Rumpf R.C., Church K.H. (2013). Broadband Microwave Frequency Characterization of 3-D Printed Materials. IEEE Trans. Compon. Packag. Manuf. Technol..

[12] Wu Y., Isakov D., Grant P.S. (2017). Fabrication of Composite Filaments with High Dielectric Permittivity for Fused Deposition 3D Printing. Materials.

[13] Tiller B., Reid A., Zhu B., Guerreiro J., Domingo-Roca R., Jackson J.C., Windmill J.F.C. (2019). Piezoelectric microphone via a digital light processing 3D printing process. Mater. Des..

[14] Isakov D., Lei Q., Castles F., Stevens C.J., Grovenor C.R.M., Grant P.S. (2016). 3D printed anisotropic dielectric composite with meta-material features. Mater. Des..

[15] Shafranek R.T., Millik C., Smith P.T., Lee C.U., Boydston A.J., Nelson A. (2019). Stimuli-responsive materials in additive manufacturing. Prog. Polym. Sci..

[16] Melchels F., Feijen J., Grijpma D. (2010). A Review on Stereolithography and Its Applications in Biomedical Engineering. Biomaterials.

[17] Choi J.S., Kang H.W., Lee I.H., Ko T.J., Cho D.W. (2009). Development of Micro-Stereolithography Technology Using a UV Lamp and Optical Fiber. Int. J. Adv. Manuf. Technol..

[18] Pham D.T., Ji C. (2000). Design for Stereolithography. Proc. Inst. Mech. Eng. Part C J. Mech. Eng. Sci..

[19] Ghoshal S. (2017). Polymer/Carbon Nanotubes (CNT) Nanocomposites Processing Using Additive Manufacturing (Three-Dimensional Printing) Technique: An Overview. Fibers.

[20] Fu K., Yao Y.Y., Dai J.Q., Hu L.B. (2017). Progress in 3D Printing of Carbon Materials for Energy Related Applications. Adv. Mater..

[21] Jin Y.F., Liu C.C., Chai W.X., Compaan A., Huang Y. (2017). Self-Supporting Nanoclay as Internal Scaffold Material for Direct Printing of Soft Hydrogel Composite Structures in Air. ACS Appl. Mater. Interface.

[22] Zhai X.Y., Ma Y.F., Hou C.Y., Gao F., Zhang Y.Y., Ruan C.S., Pan H.B., Lu W.J., Liu W.G. (2017). 3D-Printed High Strength Bioactive Supramolecular Polymer/Clay Nanocomposite Hydrogel Scaffold for Bone Regeneration. ACS Biomater. Sci. Eng..

[23] Gonzalez G., Chiappone A., Roppolo L., Fantino E., Bertanna V., Perrucci F., Scaltrito L., Pirri F., Sangermano M. (2017). Development of 3D Printable Formulations Containing CNT with Enhanced Electrical Properties. Polymer.

[24] Weng Z., Zhou Y., Lin W., Senthil T., Wu L. (2016). Structure-property relationship of nano enhanced stereolithography resin for desktop SLA 3D printer. Compos. Part A Appl. Sci. Manuf..

[25] Lin D., Jin S., Zhang F., Wang C., Wang Y., Zhou C., Cheng G.J. (2015). 3D stereolithography printing of graphene oxide reinforced complex architectures. Nanotechnology.

[26] Manapat J., Mangadlao J., Tiu B., Tritchler G., Advincula R. (2017). High-strength stereolithographic 3D Printed nanocomposites: Graphene oxide metastability. ACS Appl. Mater. Interfaces.

[27] Sandoval H., Wicker R.B. (2006). Functionalizing stereolithography resins: Effects of dispersed multi-walled carbon nanotubes on physical properties. Rapid Prototyp. J..

[28] Zhang Y.Y., Li H.M., Yang X., Zhang T., Zhu K.Q., Si W., Liu Z.L., Sun H.J. (2016). Additive Manufacturing of Carbon Nanotube-Photopolymer Composite Radar Absorbing Materials. Polym. Compos..

[29] Otter W.J., Ridler N.M., Yasukochi H., Soeda K., Konishi K., Yumoto J., Kuwata-Gonokami M., Lucyszyn S. (2017). 3D printed 1.1 THz waveguides. Electron. Lett..

[30] Fullager D.B., Park S., Hovis C., Li Y., Reese J., Sharma E., Lee S., Evans C., Boreman G.D., Hofmann T. (2019). Metalized Poly-methacrylate Off-Axis Parabolic Mirrors for Terahertz Imaging Fabricated by Additive Manufacturing. Int. J. Infrared Millim. Waves.

[31] Duangrit N., Hong B., Burnett A.D., Akkaraekthalin P., Robertson I.D., Somjit N. (2019). Terahertz Dielectric Property Characterization of Photopolymers for Additive Manufacturing. IEEE Access.

[32] Xin H., Liang M. (2017). 3-D-Printed Microwave and THz Devices Using Polymer Jetting Techniques. Proc. IEEE.

[33] Kotz F., Arnold K., Bauer W., Schild D., Keller N., Sachsenheimer K., Nargang T.M., Richter C., Helmer D., Rapp B.E. (2017). Three-dimensional printing of transparent fused silica glass. Nature.

[34] Krupka J., Gregory A.P., Rochard O.C., Clarke R.N., Riddle B., Baker-Jarvis J. (2001). Uncertainty of complex permittivity measurements by split-post dielectric resonator technique. J. Eur. Ceram. Soc..

[35] Nicholson A.M., Ross G.F. (1970). Measurement of the intrinsic properties of materials by time-domain techniques. IEEE Trans. Instrum. Meas..

[36] Weir W.B. (1974). Automatic measurement of complex dielectric constant and permeability at microwave frequencies. Proc. IEEE.

[37] Loryuenyong V., Jarunsak N., Chuangchai T., Buasri A. (2014). The photocatalytic reduction of hexavalent chromium by controllable mesoporous anatase TiO_2_ nanoparticles. Adv. Mater. Sci. Eng..

[38] Castro-Beltrán A., Luque P.A., Garrafa-Gálvez H.E., Vargas-Ortiz R.A., Hurtado-Macías A., Olivas A., Almaral-Sánchez J.L., Alvarado-Beltrán C.G. (2018). Titanium butoxide molar ratio effect in the TiO_2_ nanoparticles size and methylene blue degradation. Optik.

[39] Ponce M.A., Ramirez M.A., Schipani F., Joanni E., Tomba J.P., Castro M.S. (2015). Electrical behavior analysis of n-type CaCu_3_Ti_4_O_12_ thick films exposed to different atmospheres. J. Eur. Ceram. Soc..

[40] Ahmadipour M., Ain M.F., Ahmad Z.A. (2016). Fabrication of resistance type humidity sensor based on CaCu_3_Ti_4_O_12_ thick film. Measurement.

[41] Gong Z., Ostwald C., Hoinka N.M., Mohr B., Fuhrmann-Lieker T., Lorenz A. Ferroelectric particles in LC and polymer composite test devices. Proc. SPIE 11092, Liquid Crystals XXIII, 110920U.

[42] Mutsuddy B.C., Lee B.I., Pope E.J.A. (1994). Rheology and mixing of ceramics mixtures used in plastic molding. Chemical Processing of Ceramics.

[43] Hinczewski C., Corbel S., Chartier T. (1998). Stereolithography for the fabrication of the three-dimensional ceramic parts. Rapid Prototyp. J..

[44] White J.L., Crowder J.W. (1974). The influence of carbon black on the extrusion characteristics and rheological properties of elastomers: Polybutadiene and butadiene–styrene copolymer. J. Appl. Polym. Sci..

[45] Han C.D. (1981). Multiphase Flow in Polymer Processing.

[46] Faitel’son L.A., Alekseenko A.I. (1977). Effect of filler on the viscosity and viscoelasticity of melts of low-density polyethylene. Polym. Mech..

[47] Starr F.W., Douglas J.F., Glotzer S.C. (2003). Origin of particle clustering in a simulated polymer nanocomposite and its impact on rheology. J. Chem. Phys..

[48] Komissarenko D.A., Sokolov P.S., Evstigneeva A.D., Shmeleva I.A., Dosovitsky A.E. (2018). Rheological and Curing Behavior of Acrylate-Based Suspensions for the DLP 3D Printing of Complex Zirconia Parts. Materials.

[49] Gojzewski H., Sadej M., Andrzejewska E., Kokowska M. (2017). Dataset for acrylate/silica nanoparticles formulations and photo-cured composites: Viscosity, filler dispersion and bulk Poisson׳s ratio. Data Brief.

[50] Rao Y., Qu J., Marinis T. (2000). A precise numerical prediction of effective dielectric constant for polymer ceramic composites based on effective medium theory. IEEE Trans. Compon. Packag. Technol..

[51] An Y.J., Okino H., Yamamoto T., Ueda S., Deguchi T. (2006). Microwave Dielectric Properties of Lossy Dielectric Composite Materials. Jpn. J. Appl. Phys..

[52] Maensiri S., Thongbai P. (2007). Giant dielectric permittivity observed in CaCu_3_Ti_4_O_12_/(Li,Ti) CaCu_3_Ti_4_O_12_/(Li,Ti)-doped NiO composites. Appl. Phys. Lett..

[53] Krohns S., Lunkenheimer P., Kant C., Pronin A.V., Brom H.B., Nugroho A.A., Diantoro M., Loidl A. (2009). Colossal dielectric constant up to gigahertz at room temperature. Appl. Phys. Lett..

[54] Li Y., Li W., Du G., Chen N. (2017). Low temperature preparation of CaCu_3_Ti_4_O_12_ ceramics with high permittivity and low dielectric loss. Ceram. Int..

[55] Manchado M.A., Valentini L., Biagiotti J., Kenny J.M. (2005). Thermal and mechanical properties of single-walled carbon nanotubes–polypropylene composites prepared by melt processing. Carbon.

[56] Badev A., Abouliatim Y., Chartier T., Lecamp L., Lebaudy C., Delage C. (2011). Photopolymerization kinetics of a polyether acrylate in the presence of ceramic fillers used in stereolithography. J. Photochem. Photobiol. A.

[57] Çaykara T., Güven O. (1998). Effect of filler type on the thermal degradation of inorganic filled poly (2-hydroxyethylmethacrylate) composites. Polym. Degrad. Stabil..

[58] Jones D.I.G. (2001). Handbook of Viscoelastic Vibration Damping.

[59] Pozuelo J., Baselga J. (2002). Glass transition temperature of low molecular weight poly(3-aminopropyl methyl siloxane). A molecular dynamics study. Polymer.

[60] Das R., Kumar R., Banerjee S.L., Kundu P.P. (2014). Engineered elastomeric bio-nanocomposites from linseed oil/organoclay tailored for vibration damping. RSC Adv..

